# Novel biomarkers for early targeted and individualized treatment in Juvenile Idiopathic Arthritis

**DOI:** 10.31138/mjr.31.2.230

**Published:** 2020-06-01

**Authors:** Artemis Koutsonikoli, Anna Taparkou, Polyxeni Pratsidou-Gkertsi, Vassiliki Sgouropoulou, Theodoros Dimitroulas, Maria Trachana

**Affiliations:** 1 Paediatric Immunology and Rheumatology Referral Centre, 1^st^ Paediatric Department, Aristotle University of Thessaloniki, Hippokration General Hospital, Thessaloniki, Greece; 2 4^th^ Department of Internal Medicine, Aristotle University of Thessaloniki, Hippokration Hospital, Thessaloniki, Greece

**Keywords:** Juvenile idiopathic arthritis, PD-1 pathway, Bregs, biomarkers, immune checkpoints

## Abstract

**Background:**

The programmed cell death protein-1 (PD-1) and its ligands (PD-L 1 and 2) suppress immune responses, thus promoting self-tolerance. Among the immunomodulatory cells, acting through the PD-1 pathway, are the B-regulatory cells (Bregs). The role of the PD-1 pathway in Juvenile Idiopathic Arthritis (JIA) has not been adequately studied.

**Aims of the study:**

To investigate the immunophenotypic profile of T- and B-cells and the activity of the PD-1 pathway in JIA patients. More specifically, we will examine the levels of: a) the soluble form of PD-1 (sPD-1), b) Bregs; and the expression levels of: c) PD-1 on CD4+ and CD8+ T-cells, d) PD-L1 on Bregs and CD19+ B-cells, in blood and synovial fluid samples, at various stages of the disease (onset, relapse, remission, on or off treatment). The above biomarkers will be investigated for correlation with JIA activity.

**Methods:**

A case-control study of JIA patients (expected number: 60) and healthy controls (n: 20). Total expected number of samples: 100 of peripheral blood, 120 of serum (solely for soluble markers) and 60 of synovial fluid. The patients’ demographic data and treatment will be recorded. JIA will be classified according to the ILAR and the recently proposed PReS/PRINTO criteria. JIA activity will be assessed using the JADAS-10 tool. The biomarkers will be determined using multiparametric-polychromatic flow cytometry (quintuple fluorescence protocol) and immunoenzymatic assay ELISA.

**Anticipated benefits:**

Further elucidation of the immunophenotypic expression and variation of the abovementioned molecules and cells during active inflammation and remission in JIA. Thereby, the present study is expected to contribute to: a) the modern research and understanding of the confirmed immune dysfunction at the cellular level, which leads to the development of serious autoimmune diseases in childhood, such as JIA, and b) the search for biomarkers that could be targets of early “intelligent” treatment and thereby could support the implementation of precision-medicine. The early diagnosis and targeted treatment of JIA are crucial for the maintenance of normal physical functioning and the psychosocial balance of the still growing adolescent/child.

## BACKGROUND/INTRODUCTION

Juvenile Idiopathic Arthritis (JIA) is the most common childhood rheumatic disease. It is a heterogeneous - in respect to disease course and outcome - chronic auto-immune disease. Early diagnosis and targeted treatment are essential for the physical and psychosocial well-being of the growing child-adolescent.^1^

Recently, research has been focused on the regulation of immune checkpoints, in an attempt to elucidate the immunopathogenesis of several autoimmune diseases and consequently develop novel therapeutic targets. Immune checkpoint regulators are inhibitory or stimulatory pathways, crucial in maintaining self-tolerance. Among them is the Programmed cell Death protein-1 (PD-1) pathway.^2^ PD-1 is a cell surface protein, encoded by the PDCD1 gene, exerting its activity by binding to its two ligands (L), PD-L1 (B7-H1, CD274) and PD-L2 (B7-DC, CD273). PD-1 is expressed following antigen receptor and cytokine signals, during cell differentiation in the thymus and on various peripheral hematopoietic cells. It is expressed on immature double negative thymocytes (CD4- CD8-) and on peripheral CD4+ and CD8+ T-cells, natural killer cells, B-cells, monocytes and on some dendritic cells (DCs) upon activation. Induction of its expression occurs through signals from the T- and B-cell receptors (TCR and BCR, respectively) and is maintained high during persistent antigenic stimulation. Interleukins (ILs) 2, 7, 15, 21, which have a key role in T-cell proliferation and survival, induce PD-1 expression on T-cells. As for the ligands, PD-L1 is expressed on hematopoietic and various non-hematopoietic cells, as the vascular endothelial cells. It is constitutively expressed on B-cells, DCs, macrophages and T-cells. PD-L2 is expressed on DCs, macrophages and memory B-cells, following though a trigger-dependent induction and has a threefold binding affinity to PD-1 as compared to PD-L1. Both ligands’ expressions are regulated by the microenvironment of each inflammatory process.^3^

The PD-1 pathway transfers inhibitory signals, by exerting critical inhibitory effects during persistent antigenic stimulation (chronic viral infections, neoplastic processes, autoantigens). It is involved in regulating the immune response against self-cells, enhancing the development and function of T-regulatory cells (Tregs) and suppressing potentially pathogenic auto-reactive T-cells, thereby promoting self-tolerance. Activation of the pathway inhibits T-cell proliferation, cytokine production, cytolytic activity and disrupts their survival. At the same time, high levels of the soluble form of PD-1 (soluble PD-1, sPD-1) may reverse the immunosuppressive activity of the PD-1 pathway, by competing with the cell surface PD-1 for the binding to its ligands.^3^

Another important immune regulator is a subset of B-cells with immunomodulatory activities, the B-regulatory cells (Bregs). They suppress the differentiation of T-cells into inflammatory T-helper-1 (Th1) and Th17 cells, while promoting the recruitment of Tregs.^4^ Although the PD-1 pathway function has been studied more thoroughly in T-cells, it has been shown that one of the B-cell action pathways is through the expression of regulatory molecules, such as PD-L1.^3^

These findings led to the study of the PD-1 pathway contribution in malignancies and several autoimmune diseases, as type 1 diabetes mellitus, multiple sclerosis, idiopathic inflammatory bowel disease and Rheumatoid Arthritis (RA).

In respect to RA, there is an increased expression of PDL1 and PD-1 on T-cells (CD4+ and CD8+), monocytes and macrophages, that infiltrate the synovial tissue.^5,6^ Additionally, during the progression of undifferentiated arthritis to RA, genetics have demonstrated down-regulation of the PD-1 pathway.^6^ Interestingly, CD4+ T-cells of RA patients appear resistant to PD-1-induced suppression.^7^ sPD-1 has been found to compete with cell-surface PD-1 for binding to PD-L1, possibly inhibiting the actions induced by the binding of the two cell-surface molecules.^5,8^ High sPD-1 serum and synovial fluid levels have been identified at various stages of RA development and have been reported to correlate with disease activity, with rheumatoid factor (RF), anti-cyclic citrullinated peptide antibodies and IL21.^5–9^

These data highlight the potential importance of the PD-1 pathway in inducing autoimmune inflammation in RA and the possible use of sPD-1 as a biomarker of disease activity and a therapeutic target.

Focusing on JIA, data on the PD-1 pathway are very limited. A decreased expression of PD-L1 on myeloid cells in systemic JIA (sJIA) patients, compared with patients with febrile disease of other aetiologies, has been observed.^10^ A subsequent study reported reduced numbers of circulating peripheral blood CD4+ T-cells with reduced PD-1 expression in sJIA patients, compared to healthy controls and patients with polyarticular disease, or enthesitis-related arthritis. In addition, sJIA patients showed increased numbers of myeloid DCs, characterized by decreased PD-L1 expression, compared to healthy controls. The PD-1 expression on CD4+ T-cells of sJIA patients was negatively correlated with the number of affected joints, whereas the lowest PD-L1 expression on DCs was observed in febrile patients. These findings may be associated with the distinct clinical manifestations of sJIA patients.^11^

Regarding B-cells, a recent study in RA patients reported low PD-L1+ B-cells and PD-L1+ Bregs that increased post-treatment. Moreover, the B-cell PD-L1 expression could be in vitro modified, thus presuming the PD-L1+ B-cells as targets of future therapies.^12^

As for JIA, a recent study reported a reduced Bregs peripheral production, more pronounced in the synovial fluid, potentially contributing to the local inflammation. In particular, the number of Bregs producing IL10 was significantly lower in active disease than in remission.^13^ To our knowledge, the role of the PD-L1 expression on B-cells has not been reported.

## AIMS OF THE STUDY

Taking into account: a) the increasing interest of research in the regulation of immune system checkpoints in autoimmune diseases, b) the inadequate data on the paediatric population with JIA, and c) the importance of finding new biomarkers-therapeutic targets for personalised treatment and improving outcome of JIA, the research question of the immunomodulatory role of the PD-1 pathway in JIA has been raised.

Therefore, the aim of the present study is the investigation of the PD-1 pathway activity in JIA, by examining the immunophenotypic expression on T- and B-cells or serum concentration of the involved molecules, during active inflammation and remission.

We will determine: a) the levels of sPD-1, b) the PD-1 expression levels on CD4+ and CD8+ T-cells, c) the Bregs levels, and d) the PD-L1 expression levels on Bregs and total CD19+ B-cells, in the peripheral blood and synovial fluid of JIA patients at various stages of the disease (onset, relapse, remission, on or off treatment). In addition, these markers will be associated with the disease activity.

## PATIENTS AND METHODS

### Patients

We will conduct a case-control study of JIA patients and healthy controls. We will include: a) patients with a recent diagnosis of JIA prior to initiation of treatment (n=20), b) patients with confirmed JIA in relapse, on/off medication (n=20), c) JIA patients with available stored biological samples in our Immunology laboratory’s Biobank (n=20), and d) healthy controls (n=20).

Patients with JIA will fulfil the ILAR (International League of Associations for Rheumatology) criteria while the JIA subtypes will be classified according to the established ILAR criteria as well as the recently proposed PReS/PRINTO (Paediatric Rheumatology Society/Paediatric Rheumatology International Trials Organization) criteria.^14,15^

All participating patients will be followed in the Paediatric Immunology and Rheumatology Reference Centre (PIRRC) of the 1^st^ Paediatric Clinic of the Aristotle University of Thessaloniki, where a large number of patients with rheumatic and autoimmune diseases from different parts of Greece are monitored.

Healthy controls will be age- and gender-matched to the patients, with a recent normal immunology work-up at the PIRRC Immunology Laboratory.

### Methods

A peripheral blood sample will be collected from each group A and B patient, as part of their required immunological testing. At the same time, in patients who will need intra-articular corticosteroid infusion, for arthritis treatment, with simultaneous removal of synovial fluid prior to infusion, a synovial fluid sample will be collected. If possible, a subsequent second sample of peripheral blood, during disease remission, will be also collected, after a reasonable period of 3–6 months from the initial sample.

A sample of peripheral blood will be collected from each healthy control (group D).

Each blood and synovial fluid sample will be divided. One portion will be used for the immediate identification of cell populations, while the remainder will be centrifuged and stored at −80 ° C for the subsequent determination of soluble biomarkers.

For the determination of soluble markers, samples of serum and synovial fluid, of our Immunology Biobank will also be retrospectively studied. It is therefore estimated that approximately 20 additional stored serum and 20 synovial fluid samples will be included for the determination of sPD-1 (group C).

In total, 100 blood, 120 serum and 60 synovial fluid samples will be studied.

The demographic data of all study participants will be recorded. Clinical and laboratory findings (such as RF, anti-nuclear antibodies, inflammation markers and complete blood count) and received treatment at times corresponding to a blood/synovial fluid sample will also be recorded in every JIA participant.

Disease activity in each time point corresponding to a study sample will be assessed using the JADAS-10 tool (Juvenile Arthritis Disease Activity Score-10).^16^

Along with the biomarkers under investigation, IL17, which has already been identified as an indicator of JIA activity, will also be determined in the collected samples.^17^ Prior to inclusion in the study, an informed consent from each participant/parent will be received, according to the Helsinki Declaration.

The investigation of PD-1 expression on peripheral blood CD4+ and CD8+ T-cells will be performed by multiparametric-polychromatic flow cytometry (quintuple fluorescence protocol). Bregs will also be studied by flow cytometry in peripheral blood and synovial fluid as CD19+ CD5+ CD24hi CD38hi CD1dhi. PD-L1 expression will be studied on CD19+ B-cells and on Bregs by a combination of six fluorochromes. Monoclonal CD279 and CD274, respectively, bound to the appropriate fluorochromes will be used to determine PD-1 and PD-L1, respectively. Immunoenzymatic ELISA techniques will be used to determine sPD-1 and IL17.

Using appropriate statistical tests we will compare the immunophenotypic profile and sPD-1 levels: a) between patients and healthy controls, b) between blood/serum and synovial fluid, in case of simultaneous samples, c) between patients with active disease and remission, d) between patients with early JIA and those with long standing disease and e) between different JIA subtypes. In addition, the examined parameters will be statistically correlated with JIA activity using JADAS-10, with patients’ clinical characteristics as well as with IL17.

## ANTICIPATED BENEFITS

The present study is expected to contribute to: a) the modern research and understanding of the confirmed dysfunction of the immune system at the cellular level, which leads to the development of serious autoimmune diseases in childhood, such as JIA, and b) the search for biomarkers that could be targets of early “intelligent” treatment and thereby could support the implementation of precision-medicine.

## ABBREVIATIONS

BCR: B-Cell Receptor
Bregs: B-regulatory cells
DCs: Dendritic Cells
IL: Interleukin
ILAR: International League of Associations for Rheumatology
JADAS-10: Juvenile Arthritis Disease Activity Score-10
JIA: Juvenile Idiopathic Arthritis
L: Ligand
PD-1: Programmed cell Death protein-1
PIRRC: Pediatric Immunology and Rheumatology Referral Center
PReS: Pediatric Rheumatology European Society
PRINTO: Pediatric Rheumatology INternational Trials Organization
RA: Rheumatoid Arthritis
RF: Rheumatoid Factor
sJIA: systemic Juvenile Idiopathic Arthritis
sPD-1: soluble Programmed cell Death protein-1
TCR: T-Cell Receptor
Th: T-helper
Tregs: T-regulatory cells
